# Increasing undergraduate nursing students’ cultural competence: an evaluation study

**DOI:** 10.1186/s41256-018-0062-2

**Published:** 2018-03-05

**Authors:** Wenjia Liu, Teresa E. Stone, Rosanna McMaster

**Affiliations:** 1 School of Health Sciences, Wuhan University, No.115 Donghu Road, Wuhan, 430071 China; 2 Chiang Mai University, Chiang Mai, Thailand; 3 Professor of International Nursing, Faculty of Health Sciences, Graduate School of Medicine, Yamaguchi University, Yamaguchi, Japan

**Keywords:** Cultural competency, Educational intervention, Health disparities, Nursing students, Transformative learning

## Abstract

**Background:**

Cultural competence has become increasingly important for Chinese health professionals because of internationalization and the opening up of China to overseas visitors and business as well as a growing awareness of the needs of minority groups within China. This study aimed to evaluate a workshop designed to improve cultural competence among Chinese undergraduate nursing students.

**Methods:**

A one-group pretest and posttest design was applied. The intervention was a one-day workshop based on transformative learning theory using a variety of teaching strategies. Forty undergraduate nursing students from a university in Wuhan, China selected by convenient sampling received the intervention. Data were collected before the intervention (T1), immediately after the intervention (T2), and 1 month (T3) and 3 months (T4) following the intervention through the Chinese version of Cultural Competence Inventory for Nurses (CCIN). A researcher-designed evaluation form including open-ended questions was also used.

**Results:**

Participants’ scores by CCIN increased significantly in the total score (*p* < .001) as well as the components of cultural awareness (*p* = .003), cultural knowledge (*p* < .001), cultural understanding (*p* = .007) and cultural skills (*p* < .001), but not in cultural respect. This improvement maintained at T3 and T4. Overall, participants were satisfied with the workshop, and the qualitative results supported the effects of this intervention.

**Conclusions:**

The one-day workshop was effective in improving nursing students’ cultural competence. Replication or further refinement of this workshop is recommended for future research among additional nursing students with diverse backgrounds.

**Electronic supplementary material:**

The online version of this article (10.1186/s41256-018-0062-2) contains supplementary material, which is available to authorized users.

## Background

In this increasingly globalized world, immigration for a better standard of living, travel, study or political shelter has exposed nurses to clients with diverse cultural backgrounds. In this context, China is experiencing an increasing number of people from overseas. Based on the Census 2010 of China data, 1448 foreigners became Chinese nationals [1]. In 2014, inbound foreign tourists amounted to 26,360,000 [2]. Additionally, there are 317,098 refugees from Vietnam and an undetermined number of refugees from North Korea [3].

China itself has diversity in culture, religion and languages. Besides the dominant Han Chinese, China has fifty-five ethnic minorities including Zhuang, Hui, Manchu, Uighur, Miao, Yi, Tujia, Tibetan, Mongol, Dong, Buyei, Yao, Bai, Korean, Hani, Li, Kazakh and Dai. The percentage of Chinese people identifying with a religion were 18.2% Buddhist, 5.1% Christian, 1.8% Muslim, 21.9% folk religion, 52.2% unaffiliated and others [3]. Also, the vast variety of dialects (such as Cantonese, Minbei, Minnan, Xiang, Gan, Hakka dialects) create challenges for communication. For example, nurses from other provinces report a communication barrier when they go to work in Guangdong province, because they do not understand Cantonese, the dialect in Guangdong [4].

Despite the rapid economic growth which has released many people from poverty, it also leads to increasing disparities between those who benefited from economic advantages and those disadvantaged [5]. Health disparity remains a major problem in China, and differences exist between urban and rural areas, regions, migrants and permanent residents [5]. For example, the under-five mortality rate in urban areas was 10.7 per 1000 live births, while in rural areas it was 25.7; the maternal mortality ratio was around 3.2 times higher in rural areas than that in urban areas [6]. Within ethnic groups, the Han account for approximately 90% of the population, live in nearly every part of China and hold the most material and technical resources, while the ethnic minorities usually reside in poorer remote areas [7]. Research has shown that the minorities suffer from poorer health and nutrition status compared to the Han [8]. In addition, vulnerable populations such as people living with HIV/AIDS or mental health problems face serious discrimination and stigma from the public and even from health professionals which also contributes to health and health care disparities [9, 10].

Increasing international communication, diverse cultural backgrounds, as well as health disparities make it imperative for nurses in China to develop cultural sensitivity and competence, so that people from diverse cultural groups within and outside China can receive equal, high-quality, and culturally congruent healthcare.

Since Leininger’s work [11] on transcultural nursing in the 1960s, culturally sensitive care has become increasingly important with globalization. The concept of cultural competence has been defined from various perspectives [12], and there is yet no agreed uniform definition [13]. In this study, a simplified definition which combined the definitions given by American Academy of Nursing Expert Panel and the Transcultural Nursing Society [14, 15] was used: Cultural competence refers to the knowledge (general and specific), understanding, and skills necessary to provide acceptable, safe, patient and family centered cultural care. It involves accepting and respecting differences and not allowing personal belief to have an undue influence on those whose worldview differs from one’s own.

Cultural competence in healthcare is considered an essential goal to eliminate health-related disparities across diverse patient groups, particularly specific vulnerable populations experiencing inequities in health and healthcare [16]. Health outcomes differ from the mainstream population for ethnic minorities, the underserved, underrepresented and other vulnerable populations who are disproportionately afflicted by diseases, injuries and disorders. While demographic, socioeconomic or geographic strata account for the disparities; this is compounded by the suboptimal health care provided by health professionals due to lack of cultural competence [14, 17]. Thus, improving cultural competence of healthcare providers is crucial to address health disparities [14, 18].

A person’s ethnic and socio-cultural background can affect their perceptions of health and willingness to receive health care [19]. Failure to recognize, understand and manage a patient’s sociocultural variations may impede effective communication, lead to mistrust, patient dissatisfaction, non-adherence, and poorer health outcomes [20]. Therefore, developing cultural competency would be beneficial to promote client-caregiver relationship and improve health outcomes.

Substantial empirical literature has identified that education interventions could improve health providers’ cultural competence in aspects of knowledge, attitudes and skills, irrespective of the differences in duration, design or contents of the interventions, although most studies were low to moderate in the degree of quality [16, 21–23]. A variety of teaching strategies were reported, including traditional lecture, demonstration, case-based learning, small group activity, role-play, and self-exploratory exercises. Teaching formats encompassed workshops, seminars, university courses, and immersion in a different culture [12, 24].

However, compared to developed countries, China’s progress in developing cultural competence is still in its infancy [4, 25]. In most medical colleges, cultural competence education has not been included in the required courses for nursing students. Even those colleges offering cultural competence education only include related content as a small part in an introductory course and this cannot fully achieve the goal of improving nursing students’ cultural competence. [26]. Papers from China have only contributed to 0.3% of global research in this area [27]. Most studies are limited to reviews and investigations, few studies concern practical intervention [28]. Moreover, traditional didactic teaching in class was still the dominant pedagogical method, and an absence of a variety of flexible teaching strategies was noticeable [29]. Therefore, conducting interventions to improve nurses’ and nursing students’ cultural competence with multifaceted pedagogical strategies is imperative in China.

### Pedagogical framework

Transformative Learning Theory developed by Mezirow [30] was the theoretical framework underpinning the teaching in this workshop. Mezirow proposed “perspective transformation”: the process of becoming critically aware of how and why our assumptions influence our perspectives and develop a more inclusive perspective by changing these habitual constructs. People tend to reject ideas that violate their existing cognitive system, and these conflicting ideas produce opportunities for transformation and growth, resulting in either intensification of previously held beliefs or some degree of transformation [31, 32]. Transformation can be realized by critical reflection, including being critically reflective of one’s own assumptions to change the taken-for-granted frame of reference, and of others’ assumptions that lead to successful communication. Fostering emancipated, autonomous and responsible thinkers is the ideal outcome of transformative learning [33].

There are three major phases of the Transformative Learning Theory. This process is sparked by a “disorienting dilemma” [30] (p. 168), described by Cranton [34] as a “catalyst for transformation”, “an activating even that typically exposes a discrepancy between what a person has always assumed to be true and what has just been experienced, heard or read” (p. 66). Next, learners engage in critical reflection and reevaluate the assumptions they hold about themselves and the world. They develop new perspectives to look at their practice and revise their views [33, 35]. Then, they move to “reflective discourse” [36] (p. 11), sharing the new views with other people to obtain consensual validation. Finally, they change their behavior based on the new perspective [37].

## Methods

### Aim

The aim of this study was to conduct a designed one-day workshop and evaluate its immediate and long-term effects among undergraduate nursing students in China on improving their cultural competence.

### Design

This study employed a one-group pretest and posttest design. Participants were invited to attend a one-day cultural competence workshop given by the researcher in groups of eight to twelve. The teaching strategies included condensed didactic lectures [38] and a series of self-reflective activities such as video vignettes watching, drawing cultural self-portrait as well as Social Attitude Implicit Association Test [39, 40]. In addition, concept matching, a cross-cultural simulation game *Barnga* and debriefing [41], a documentary about people living with HIV/AIDS [42] and small group reflective discussions, clinical culture care case studies [43] and role play were undertaken. Details of this workshop schedule and teaching materials can be found in the additional files [see Additional files 1 and 2].

Data were collected 2 weeks before the workshop (T1), immediately after the workshop (T2) and at 1 month (T3) and 3 months (T4) following the workshop to evaluate the short-term and long-term effects of the intervention.

### Sample

Undergraduate nursing students at a University in Wuhan, China were invited to participate in this study by convenience sampling. The sample size was determined by power analysis using G-Power software. Set the moderate effect size = 0.5, power = 0.80, and alpha = 0.05, the minimum sample size was calculated as 34.

### Instruments

The Chinese version of Cultural Competence Inventory for Nurses (CCIN) developed by Dr. Duanying Cai of Chiang Mai University [44] was used with permission to assess nursing students’ cultural competence. CCIN consists of 29 items including five dimensions: cultural awareness, cultural respect, cultural knowledge, cultural understanding and cultural skills. Responses to these items are rated using a 5-point Likert scale (1 = not at all/strongly disagree, 2 = occasionally/disagree, 3 = sometimes/uncertain, 4 = often/agree, 5 = always/strongly agree). Correspondingly, each item was scored from 1 (never/strongly disagree) to 5 (always/strongly agree), thus the total scores range from 29~ 145. The Cronbach’s alpha of the overall scale was .94, and for the individual dimensions it ranges from .79 to .92, indicating high internal consistency. The validity was also tested by the developer. Completion time for this questionnaire was approximately 10 min. In addition, a demographic variables questionnaire was given to the participants together with the pretest, and an evaluation form including fourteen 5-point Likert scale questions (5 = very satisfied, 4 = satisfied, 3 = neutral, 2 = unsatisfied, 1 = very unsatisfied) and three open-ended questions was given immediately after the workshop to evaluate participants’ degree of satisfaction and gains. Open-ended questions included: “What did you learn that you had not been aware of before this workshop?”, “What will you do differently to nurse people from other cultures in the future?” and “What suggestions do you have for improving this workshop?”

### Data analysis

The demographic variables were analyzed by frequency distributions and percentages. Mean, standard error of mean (SEM), median, standard deviation (SD), and range were used for describing the CCIN scores across the four time points. Paired t-test or Wilcoxon signed ranks test was used to examine the CCIN score differences between pretests and immediate posttest separately on the total score and each of the components based on the distribution. A repeated-measured ANOVA was conducted to compare the means of CCIN total scores at all time points. Differences of changes in CCIN total scores (post-test minus pre-test) in different baseline demographic characteristics were also examined using analysis of variance (ANOVA) or nonparametric test based on the distribution. The IBM SPSS Statistics 22.0 was used for all the statistical analyses and statistical significance was set at *p* < .05. Content analysis was conducted to analyze the written responses to the open-ended questions in the evaluation form, based on the process suggested by Graneheim and Lundman [45].

## Results

A total of forty undergraduate nursing students completed the pre-test, attended the intervention, completed the post-test and the one-month and three-month follow-ups. Table 1 presents the demographic results. The average age of the participants was 20 years, and the majority were female. Over half the participants were from Hubei province, where the university study site is located. Most reported not following a religion, while one student identified as Buddhist, and two as Protestants. Thirty-three (82.5%) were Han Chinese and seven (17.5%) belonged to ethnic minorities of China. Eleven (27.5%) were first year students, seven (17.5%) were second year, eight (20%) third year, and 14 (35%) in their fourth and final year. One quarter were from classes taught in Chinese; the others were from international classes taught in English. A vast majority had no previous overseas travel experience although many of them (77.5%) were fond of travelling. Most of them had passed the standard Chinese college English test, and one quarter had learned a second foreign language. Only one participant had undertaken culture-related training before, but most were exposed to diverse cultures via websites, newspapers, television or other media to some degree. Regarding clinical nursing practice, 15 (37.5%) students had practiced nursing for less than 1 month, eight (20%) students had practiced less than 3 months, while 17 (42.5%) students had practiced for more than 3 months.

**Table 1:** Demographic characteristics (*N* = 40)

Characteristic	n	%
Age		
17	1	2.5
18	7	17.5
19	7	17.5
20	4	10.0
21	9	22.5
22	10	25.0
23	2	5.0
Mean (SD)	20.3 (1.7)	
Gender		
Male	7	17.5
Female	33	82.5
Home town		
In Hubei province^a^	23	57.5
In other provinces	17	42.5
Religion		
None	37	92.5
Buddhism	1	2.5
Protestantism	2	5.0
Ethnicity		
Han	33	82.5
Tujia	3	7.5
Hui	1	2.5
Miao	1	2.5
Zhuang	1	2.5
Dong	1	2.5
Grade		
1st year	11	27.5
2nd year	7	17.5
3rd year	8	20.0
4th year	14	35.0
Travel overseas		
Yes	3	7.5
No	37	92.5
No. of visited overseas countries		
0	37	92.5
1	3	7.5
English level		
CET-4 ≥ 425^b^	21	52.5
CET-6 ≥ 425^c^	18	45.0
TOEFL ≥80	1	2.5
Second foreign language		
Yes	10	25.0
No	30	75.0
Previous related training		
Yes	1	2.5
No	39	97.5
Total time of clinical nursing experience (months)		
< 1	15	37.5
1–3	8	20.0
> 3	17	42.5
Exposure to cultural diversity		
Often	17	42.5
Sometimes	15	37.5
Occasionally	6	15.0
Never	2	5.0
Travel as a hobby		
Yes	31	77.5
No	9	22.5
Type of Class		
International	30	75.0
None-international	10	25.0

Note: ^a^The university as the study setting is in Hubei province

^**b**^College English Test Band 4, a standard English level test in China with a pass mark of 425

^**c**^College English Test Band 6, a higher English level test in China with a pass mark of 425

The mean score of pretests by CCIN was 101.58 (SD = 14.53), with scores ranging from 66 to 143 (The standard score range is 29~ 145). The mean score of the posttests increased to 111.88 (SD = 15.14), with the minimum of 64 and maximum of 143. As to the follow-up scores, they kept decreasing slightly across time compared to the posttest score, but remained higher than the pretest score. The CCIN score differences between pretests and immediate posttest were examined, and significant increases were found on the total score (*p* < .001) as well as the components of cultural awareness (*p* < 0.01), cultural knowledge (*p* < .001), cultural understanding (*p* < 0.01), and cultural skills (*p* < .001). The scores in cultural respect decreased somewhat but was not statistically significant (*p* > .05) (Table 2).

**Table 2:** Comparison between pretest and immediate posttest CCIN scores (N = 40)

Component	No. of questions	Pretest Mean (SD)	Posttest Mean (SD)	*P* Value
Cultural awareness	6	23.93 (3.98)	25.98 (3.77)	.003^*^
Cultural respect	5	19.70 (2.27)	19.55 (2.26)	.625
Cultural knowledge	4	11.15 (2.92)	12.88 (2.53)	.000^*^
Cultural understanding	5	19.05 (2.90)	20.65 (3.57)	.007^*^
Cultural skills	9	27.75 (6.51)	32.83 (5.71)	.000^*^
Total score	29	101.58 (14.53)	111.88 (15.14)	.000^*^

Note: *Significant results based upon α = 0.05

**Fig. 1:**
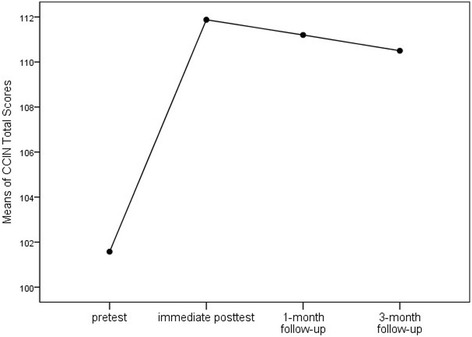
Change of average CCIN total score over time

The means of CCIN total scores at all time points were compared by repeated-measured ANOVA. The overall results were statistically significant (F = 11.835, *P* < 0.001), indicating that the participant’s scores differed over time. The following pairwise comparisons with Bonferroni adjustment found that statistically significant differences were found between the mean scores of T1 and T2 (*p* < .001), T1 and T3 (*p* < .001), as well as T1 and T4 (*p* = .001), suggesting that the cultural competence workshop was observably effective in increasing the participants’ cultural competence immediately and over time. However, the mean scores at the follow-ups decreased continually compared to the immediate posttest scores, nevertheless the differences were not significant (*p* > .05) (Table 3). The change pattern of CCIN total scores over time was demonstrated in Fig. 1.

**Table 3:** Repeated measures ANOVA for difference in of CCIN total mean scores. over the four time points (N = 40)

Measure	Pretest Mean (SD)	Posttest Mean (SD)	1-month follow-up Mean (SD)	3-month follow-up Mean (SD)	*F*	*P* Value
Total score	101.58 (14.53)	111.88 (15.14)	111.20 (14.83)	110.50 (14.71)	11.835	<.001^*^

Note: ^*^Significant results based upon α = 0.05

Differences of changes in CCIN total scores in different baseline demographic characteristics were examined. Age was not analyzed because the range was narrow and the count for some items was too few. As to religion, ethnicity, and English level, because there were too few in certain categories, some items were re-categorized into combined groups for analysis. For example, Tujia, Hui, Miao, Zhuang and Dong were combined into “other” (ethnicities except Han); English level of CET-4 ≥ 425 was defined as “basic” while CET-6 ≥ 425 and TOEFL ≥80 were combined and defined as “higher”. However, no statistically meaning results were found, perhaps due to the small sample size.

The Cronbach’s alpha of the overall aspect of the CCIN was calculated at .935 for pretest and .954 for posttest, and for the five components of CCIN it ranged from .772 to .913 for pretest and from .702 to .908 for posttest, demonstrating satisfactory reliability of this scale.

Participants’ satisfaction regarding the different teaching strategies and the overall quality of the workshop was assessed by a researcher-designed evaluation form. In summary, participants were satisfied with the overall quality of this workshop in the aspects of content, atmosphere, management and time control, with mean scores ranging from 4.48 to 4.73. Most teaching strategies were acceptable, except the concept-matching activity of which the mean score was 3.98 (Table 4).

**Table 4:** Average Ratings of the Evaluation Form for the Workshop

Item	Mean
*Teaching strategies*	
Bargna game	4.63
Video vignettes about culture	4.28
Cultural self-portrait activity	4.32
Concept matching activity	3.98
Documentary on HIV/AIDS in China	4.68
Social Attitude Implicit Association Test	4.55
Cultural scenarios case study	4.45
Reflective discussion after each activity	4.35
*Overall quality of this workshop*	
Overall satisfaction	4.48
Content	4.48
Teaching strategies	4.53
Class atmosphere	4.60
Overall management	4.73
Time management	4.40

### Open-ended responses: evaluation of the workshop

A total of forty participants responded to the open-ended questions which concerned an evaluation of the workshop. From these responses, the following factors emerged as most frequently cited from questions: new knowledge gained; change in future practice and suggested improvements to workshop.

### New knowledge gained

Two categories were identified from responses: (a) cultural competence; (b) transformative learning. Cultural competence involved learning something new in the components of cultural competence, including cultural knowledge, cultural awareness, cultural understanding, cultural sensitivity and cultural skills. Participants reported that they had gained “a lot of knowledge of nursing in transcultural field”. In addition, “differences in culture and awareness can have a huge influence, and the diversity of understanding is ubiquitous and cannot be ignored”. Other students reported that “Diverse cultural backgrounds should be given more understanding and respect.” There were also some students who “learned how to use professional skills when facing different ethnic groups.”

Transformative learning referred to the participants’ perspective being changed or transformed through reflective thinking. Some students said this workshop elicited their “unconscious thoughts”, which made them reflect upon themselves. Others said that they were “motivated to begin thinking” and realized that “something we take for granted can be looked at in totally the opposite way by other people”. Therefore, “we should avoid stereotypes and preconceived notions” and “think from patients’ perspectives.”

### Change in future practice

Participants expressed their willingness to improve cultural knowledge, cultural awareness, cultural sensitivity, and cultural skills in future practice. Statements such as “I will learn about their backgrounds first, and refer to literature, to provide better service,” “Treat some of their behaviors with understanding and tolerance,” and “I will observe and listen to the personal needs of patients from diverse cultural backgrounds more actively” reflected this category. Participants also showed the desire to be professional and confident nurses who hold reflective thinking and provide equal and cultural congruent healthcare to patients. For example, some hoped to “communicate more with the patients rather than make subjective assumptions,” and “stand in patients’ position, and provide acceptable nursing intervention”.

### Improvements to workshop

The majority of the students regarded this workshop as interesting and meaningful. For instance, one student said: “I didn’t expect a lot before coming, but this activity was beyond my expectation and brought me a lot of pleasant surprises.” Suggestions for improving the workshop included the management of time, organization, material presentation and increase of activities. Other improvements included expansion of teaching content and diversity of participants. For example, one student stated: “I hope that more students with distinct characteristics will join in this workshop.” Another student suggested that “Some other contents in transcultural field can be added, such as how to understand and learn the differences between western nursing knowledge and Chinese culture.”

## Discussion

This study aimed to evaluate the immediate and long-time effects of a designed one-day workshop on improving cultural competence among Chinese undergraduate nursing students. As anticipated, participants’ scores on cultural competence, especially in aspects of cultural awareness, cultural knowledge, cultural understanding, and cultural skills increased significantly immediately after the intervention. This is consistent with substantial previous empirical studies which affirmed the positive effects of cultural competence education in other cultural contexts through courses [24, 46, 47], workshops [48] or training [49, 50]. However, the scores on the component of cultural respect did not increase significantly after the intervention. Cultural respect in this instrument was defined as “the essential attitudes to cultural diverse clients, such as feeling comfortable in cultural encounters, treating them equally and respecting their beliefs, values and behaviors” [44]. This negative result was possibly attributable to participants’ lack of clinical practice experience in providing care for people from diverse cultures. Another possibility was that participants might have a better understanding of the meanings of the corresponding items after the workshop, which allowed them to evaluate themselves more objectively and thus decreased the scores.

In this study, the intervention lasted for around 6 h within 1 day. Education sessions in many of the other studies were conducted over more extended time periods, from several weeks [24, 51] to years [52, 53], and some study even included field immersion experience [54]. However, shorter-time interventions have also been proved successful. For example, a study in Taiwan involved two 2-h workshops and a 2-h practice session [55]. Another study included three 1-h seminars and a 3-h workshop [56]. A more recent study even proved that a 1-h training was effective to improve clinical nurses’ cultural competence for a long time [50]. This study provided further evidence that it is possible to influence cultural competence using a brief interactive educational intervention. Given the limitation of clinical nurses and nursing students’ available time, a shorter-time intervention may be more practical.

The cultural competence scores did not increase again at the one-month and three-month follow-ups, although they were still significantly (*P* ≤ .001) higher than the pretest scores. This is at odds with some previous studies. In Brathwaite’s study [24], participants’ scores increased again at 3-month follow-up. In the study of Wilson, Sanner and McAllister [48], the scores grew from pretest to posttest until 12-month follow-up. In the study of Thomas and Cohn [57], participants’ scores increased again at 3-month follow-up but stabilized at 6-month. The heterogeneity in the duration of intervention, the scales and the sample may be accountable for the differences at follow-ups. The length of follow-up also influences the results. In current study, the 3-month decline may imply that ongoing periodic education should be implemented to strengthen the memory and longer time follow-ups need to be done in the future research.

The multiple teaching strategies used in the workshop seemed to be effective in improving participants’ cultural competence. Results of the evaluation form scores and the written responses to the open-ended questions revealed participants’ overall satisfaction of the intervention. Material intended to trigger reflection - the cross-cultural simulation game *Barnga* and the documentary about people living with HIV/AIDS in China - were graded highest by the participants, while concept learning activity was graded lowest. The concept learning was not welcomed perhaps due to the amount and complexity of the given concepts and the limited time to learn and digest. Research shows that the complexity of concepts is in parallel with the difficulty of learning, and decreased complexity could increase the success of learning [58].

Simulation is increasingly being used as an experiential immersion method in cultural awareness training [41]. In simulation games, learners can practice skills that will be needed in intercultural situations by exposure to safe imitative encounters, and also learn about the culture of themselves and of others [59]. In *Barnga,* participants experienced confusion and conflicts between unfamiliar cultures, and learned to adapt to new environment. They also realized the value of language, through the strong uncomfortable feelings and the unpleasant misunderstandings due to the use of only non-verbal communication. In the subsequent debriefing, participants recalled the scenarios of adapting to cultural differences in real life, and were further aware of their hidden stereotypes and prejudices. It was obvious that this game successfully increased participants’ cultural awareness, as in the study of Koskinen, Abdelhamid and Likitalo [41]. The documentary about people living with HIV in impoverished areas of China evoked strong responses among the participants. By recalling the unequal treatment towards all kinds of vulnerable populations and discussing the underlying reasons behind people’s discrimination, an ardent desire to reduce healthcare disparities was evoked. Similar reflections were also elicited by the cultural self-portrait and Social Attitude Implicit Association Test, evincing the effects of these activities to enhance cultural awareness.

The qualitative results indicated that participants learned new knowledge about cultural competence and gained a desire to practice as a culturally competent nurse in the future. Participants also experienced perspective transformation. For example, some of the participants began to rethink cultural aspects they took for granted before the workshop and increased their awareness to avoid discriminative treatment to others. Mezirow [33] stated that transformative education should be learner-centered, participatory, and interactive. Pedagogical methods such as role play, case studies, simulations and other active learning strategies promoting the learners to connect their own experiences with new knowledge are encouraged. The results of this study confirmed the success of multiple active teaching methods for perspective transformation. Previous studies based on transformative learning also reported positive results in improving participants’ cultural competence [60] or transcultural self-efficacy [32], suggesting it was an effective theory in culture care education.

Recruiting participants who can participant in a condensed one-day workshop which lasted approximately 6 h was challenging for the cohort of college nursing students. It is also likely to be difficult to disseminate a workshop of this duration to clinical nurses. Separating the intervention into separate fragments of teaching hours might be a solution, or a blended model like a flipped classroom [61] could be successful, where students gain first exposure to new materials like reading or lecture videos outside of class in advance, with face to face time used to engage in learner-centered activities and deeper discussions. In this case, lecture documents, videos as well as the online Social Attitude Implicit Association Test could be assigned as pre-work, leaving the class time for the simulation game, debriefing and case study.

### Strengths & Limitations

Findings in this study demonstrated the positive effects of the employed teaching strategies and the pedagogical framework. The qualitative data further showed participants’ desire to be cultural competent nurses in the future. Given that most accessible good-quality teaching materials about cultural competence were from Western countries, some materials used in this study, such as the examples of health and healthcare disparities as well as the clinical culture care cases, were based on western context and not applicable for Chinese learners. In order to make the intervention more cultural appropriate, some other teaching materials were revised based on the cultural context in China, which promoted the success of this workshop to some degree. However, more culturally appropriate teaching materials suitable for Chinese or Asian learners still need to be developed.

The Chinese version of Cultural Competence Inventory for Nurses (CCIN) was applied in empirical study for the first time. Future studies using this instrument are needed to further examine the reliability and validity of this tool. The convenience sampling, small sample size and single study setting may limit the generalization of the results, and no comparison group provided potential alternative explanations for the causality of the score changes. Rigorous research with larger sample, larger scope, longer follow-up duration as well as comparisons are recommended for the future. Further, this study only used self-reported data which had a subjective nature and could be affected by social desirability bias [62, 63]. Future studies could use more objective evaluations such as observational methods and also consider patients’ outcomes.

### Implications for nursing education, practice, and research

This study using a variety of teaching strategies proved to be effective in improving Chinese undergraduate nursing students’ cultural competence. Replication or further refinement of this intervention is recommended. A tighter and more flexible teaching schedule for health professionals needs to be developed. Nursing researchers, educators and administrators are recommended to emphasis cultural competence in studies, education and policy making processes to improve patients’ health outcomes and reduce health care disparities.

## Conclusions

This one-day workshop designed under Transformative Learning Theory has shown to be effective in improving undergraduate nursing students’ cultural competence in the components including cultural awareness, cultural knowledge, cultural understanding, and cultural skills. Incorporating this workshop into college education on nursing students in China would be beneficial to prepare them to provide culturally sensitive care for increasing patients with diverse cultural backgrounds after graduation.

## Additional files

Additional file 1: Design of the one-day cultural competence workshop. (PDF 331 kb)

Additional file 2: Descriptions of the main teaching materials used in this study. (PDF 510 kb)

## Abbreviations

ANOVA: analysis of variance
CCIN: Chinese version of cultural competence inventory for nurses
CET-4: College English Test Band 4
CET-6: College English Test Band 6
HIV/AIDS: Human immunodeficiency virus infection and acquired immune deficiency syndrome
SD: standard deviation
SEM: standard error of mean
USDHHS OMH: Department of health & human services, office of minority health

## References

[1] National Bureau of Statistics of the People’s Republic of China. Tabulation on the 2010 population census of the People’s republic of China. 2010. http://www.stats.gov.cn/tjsj/pcsj/rkpc/6rp/indexch.htm. Accessed 15 Aug 2016.

[2] National Bureau of Statistics of China. National Data, 2014. http://data.stats.gov.cn/easyquery.htm?cn=C01. Accessed 15 Aug 2016.

[3] Central Intelligence Agency. The world factbook. 2016. https://www.cia.gov/library/publications/resources/the-world-factbook/geos/ch.html. Accessed 11 July 2016.

[4] Gao J, Chen PY (2015). The thought about transcultural nursing education in nursing colleges in the international situation. Chin Gen Pract Nurs.

[5] Meng Q, Zhang J, Yan F, Hoekstra EJ, Zhuo J (2012). One country, two worlds – the health disparity in China. Global Public Health.

[6] China Ministry of Health and World Health Organization. Joint review of the maternal and children survival strategy in China. China ministry of health. 2006. http://www.crin.org/en/docs/unicef_mat_child_survival.pdf. Accessed 20 Aug 2016.

[7] Wang X, Pan J. Assessing the disparity in spatial access to hospital care in ethnic minority region in Sichuan Province, China. BMC Health Serv Res. 2016; 10.1186/s12913-016-1643-8. 10.1186/s12913-016-1643-8 PMC4989300 27535827

[8] Ouyang Y, Pinstrup-Andersen P (2012). Health inequality between ethnic minority and Han populations in China. World Dev.

[9] Gao SY, Fei LP (2001). Attitudes about mental illness of different types of respondents in Beijing. Chin Mental Health J.

[10] Zhang Y, Niu WY, Sun XM (2011). The attitudes and its influential factors of health personnel towards people living with HIV/AIDS: an example of two general hospitals in Beijing. Med Philos.

[11] Leininger M. Culture care theory, research, and practice. Nurs Sci Q. 1996; 10.1177/089431849600900208. 10.1177/089431849600900208 8710313

[12] Pugh, PACU: Effective educational delivery methods: a meta-analysis of cultural competence education for health care professionals*.* http://en.whu.findplus.cn/?h=articles&db=psyh&an=2008-99100-138 (2008). Accessed 12 June 2016.

[13] Horvat L, Horey D, Romios P, Kis-Rigo J (2014). Cultural competence education for health professionals. Cochrane Database Syst Rev.

[14] Giger J, Davidhizar RE, Purnell L, Harden JT, Phillips J, Strickland O (2007). American Academy of Nursing expert panel report: developing cultural competence to eliminate health disparities in ethnic minorities and other vulnerable populations. J Transcult Nurs.

[15] Douglas MK, Rosenkoetter M, Pacquiao DF, Callister LC, Hattar-Pollara M, Lauderdale J (2014). Guidelines for implementing culturally competent nursing care. J Transcult Nurs.

[16] Gallagher RW. A meta-analysis of cultural competence education in professional nurses and nursing students. 2011.http://scholarcommons.usf.edu/etd/3112/. Accessed 7 June 2016. 10.1016/j.nedt.2014.10.021 25466790

[17] U. S. Department of Health and Human Services. The secretary's advisory committee on National Health Promotion and disease prevention objectives for 2020. Phase I report: Recommendations for the framework and format of Healthy People 2020. 2008. http://www.healthypeople.gov/sites/default/files/PhaseI_0.pdf. Accessed 10 June 2016.

[18] Kirmayer LJ. Rethinking cultural competence. Transcult Psychiatry. 2012; 10.1177/1363461512444673. 10.1177/1363461512444673 22508634

[19] Musolino GM, Babitz M, Burkhalter ST, Thompson C, Harris R, Ward RS (2009). Mutual respect in healthcare: assessing cultural competence for the University of Utah Interdisciplinary Health sciences. J Allied Health.

[20] Betancourt JR, Green AR (2010). Commentary: linking cultural competence training to improved health outcomes: perspectives from the field. Acad Med.

[21] Beach MC, Price EG, Gary TL, Robinson KA, Gozu A, Palacio A (2005). Cultural competence: a systematic review of health care provider educational interventions. Med Care.

[22] Lie DA, Lee-Rey E, Gomez A, Bereknyei S, Iii CHB (2011). Does cultural competency training of health professionals improve patient outcomes? A systematic review and proposed algorithm for future research. J Gen Intern Med.

[23] Truong M, Yin P, Priest N (2014). Interventions to improve cultural competency in healthcare: a systematic review of reviews. BMC Health Serv Res.

[24] Brathwaite AEC. Evaluation of a cultural competence course. J Transcult Nurs. 2005; 10.1177/1043659605278941. 10.1177/1043659605278941 16160199

[25] Huo M: The development of the rating scale of cultural competence and investigation on the status of cultural competence for nurses. 2009. http://scholarcommons.usf.edu/cgi/viewcontent.cgi?article=4307&context=etd. Accessed 5 June 2016.

[26] Zhang XL, Peng YQ, Yu HP, Xu L (2010). The development of multicultural nursing education in China. Chin Gen Pract.

[27] Li J, Luo Y, Xu HB, Zeng T (2015). The bibliometrics analysis of transculture nursing literature based on the web of science database. Chin Nurs Manage.

[28] Shi MM, Cao MJ (2015). Status quo of cultural competencies cultivation among undergraduate nursing students and its countermeasures. J Nurs Sci.

[29] Chen Y, Su LX, Ouyang MY, Liu GY (2014). The research progress of cultural competence education in China and overseas. Intern Med China.

[30] Mezirow J (1991). Transformative dimensions of adult learning.

[31] Taylor EW (1994). Intercultural competency: a transformative learning process. Adult Educ Q.

[32] Lonneman W. Teaching strategies to increase cultural awareness in nursing students. Nurse Educ. 2015; 10.1097/NNE.0000000000000175. 10.1097/NNE.0000000000000175 25997149

[33] Mezirow J (1997). Transformative learning: theory to practice. New Dir Adult Contin Educ..

[34] Cranton P (2002). Teaching for transformation. New Dir Adult Contin Educ.

[35] Sokol A, Cranton P (1998). Transforming, not training. Adult Learn.

[36] Mezirow J, Mezirow's JA (2000). Learning to think like an adult: Core concepts of transformation theory. Learning as transformation: critical perspectives on a theory in progress.

[37] Baumgartner LM (2001). An update on transformational learning. New Directions for Adult & Continuing Education.

[38] U. S. Department of Health & Human Services, Office of Minority Health. Culturally competent nursing care: a cornerstone of caring. n.d. https://ccnm.thinkculturalhealth.hhs.gov/Content/Course1/Module5/Module1_5_1.asp. Accessed 15 July 2016.

[39] Steed R. Attitudes and beliefs of occupational therapists participating in a cultural competency workshop. Occup Ther Int. 2010; 10.1002/oti.299. 10.1002/oti.299 20641132

[40] Project Implicit. Project implicit social attitudes. 2011. https://implicit.harvard.edu/implicit/. Accessed 24 August 2016.

[41] Koskinen L, Abdelhamid P, Likitalo H (2008). The simulation method for learning cultural awareness in nursing. Divers Health Soc Care.

[42] CCTV Official website. Eyeshot of phoenix: the documentary of HIV/AIDS in China. 2011. Retrieved from http://tv.cntv.cn/videoset/C32873 DEED5DA62CBDB8. Accessed 1 July 2016.

[43] The American College of Obstetricians and Gynecologists (2011). Committee opinion no. 493: cultural sensitivity and awareness in the delivery of health care. Obstet Gynecol.

[44] Cai D, Kunaviktikul W, Klunklin A, Sripusanapan A, Avant P (2017). Developing a cultural competence inventory for nurses in China. Int Nurs Rev.

[45] Graneheim UH, Lundman B (2004). Qualitative content analysis in nursing research: concepts, procedures and measures to achieve trustworthiness. Nurse Educ Today.

[46] Hughes KH, Hood LJ (2007). Teaching methods and an outcome tool for measuring cultural sensitivity in undergraduate nursing students. J Transcult Nurs.

[47] Cerezo PG, Galceran MS, Soriano MG, Camps LM, Moral JL. Design and evaluation of an educational course in cultural competence for nursing. 2014. http://en.whu.findplus.cn/?h=articles&db=edselp&an=S1877042814032182. Accessed 10 June 2016.

[48] Wilson AH, Sanner S, McAllister LE (2010). A longitudinal study of cultural competence among health science faculty. J Cult Divers.

[49] Berlin A, Nilsson G, Törnkvist L (2010). Research article: cultural competence among Swedish child health nurses after specific training: a randomized trial. Nurs & Health Sci.

[50] Delgado DA, Ness S, Ferguson K, Engstrom PL, Gannon TM, Gillett C (2013). Cultural competence training for clinical staff: measuring the effect of a one-hour class on cultural competence. J Transcult Nurs.

[51] Genao I, Bussey-Jones J, St. George DM, Corbie-Smith G (2009). Empowering students with cultural competence knowledge: randomized controlled trial of a cultural competence curriculum for third-year medical students. J Natl Med Assoc.

[52] Salman A, McCabe D, Easter T, Callahan B, Goldstein D, Smith TD (2007). Cultural competence among staff nurses who participated in a family-centered geriatric care program. J Nurses Staff Dev.

[53] Jeffreys MR, Dogan E (2012). Evaluating the influence of cultural competence education on students’ transcultural self-efficacy perceptions. J Transcult Nurs.

[54] Caffrey RA, Neander W, Markle D, Stewart B (2005). Improving the cultural competence of nursing students: results of integrating cultural content in the curriculum and an international immersion experience. J Nurs Educ.

[55] Ho M, Yao G, Lee K, Beach MC, Green AR (2008). Cross-cultural medical education: can patient-centered cultural competency training be effective in non-western countries?. Med Teach.

[56] Leiper J, Van Horn ER, Hu J, Upadhyaya RC (2008). Promoting cultural awareness and knowledge among faculty and doctoral students. Nurs Educ Perspect.

[57] Thomas VJ, Cohn T (2006). Communication skills and cultural awareness courses for healthcare professionals who care for patients with sickle cell disease. J Adv Nurs.

[58] Feldman J (2010). The simplicity principle in human concept learning. Curr Dir in Psychol Sci.

[59] Fowler SM, Pusch MD (2010). Intercultural simulation games: a review (of the United States and beyond). Simul Gaming.

[60] Hawala-Druy S, Hill MH (2012). Interdisciplinary: cultural competency and culturally congruent education for millennials in health professions. Nurse Educ Today.

[61] Roehl A, Reddy SL, Shannon GJ (2013). The flipped classroom: an opportunity to engage millennial students through active learning strategies. J Fam Consum Sci.

[62] Brown B, Warren NS, Brehm B, Breen P, Bierschbach JL, Smith R (2008). The design and evaluation of an Interprofessional elective course with a cultural competence component. J Allied Health.

[63] Kardong-Edgren S, Campinha-Bacote J (2008). Cultural competency of graduating US bachelor of science nursing students. Contemp Nurse.

