# Supplementary material for: Increasing undergraduate nursing students’ cultural competence: an evaluation study
Source: Glob Health Res Policy. 2018 Mar 5;3:7. doi: 10.1186/s41256-018-0062-2 (PMC5836416; doi:10.1186/s41256-018-0062-2)
Supplement: Supplementary file 1 — Design of the one-day cultural competence workshop. (PDF 331 kb) [file 41256_2018_62_MOESM1_ESM.pdf]

Additional file 1. Design of the one-day cultural competence workshop

Section	Content	Time
Section 1 – Cultural Awareness (8:00-11:30 am)		
Disorienting Dilemma	Cross-cultural simulation game: Play *Barnga*<br>Debriefing (Small Group Discussion)	90 minutes
Critical Reflection	a. What is culture?<br>Video vignettes: I. *What is Culture?* (Bureau of Educational and Cultural Affairs; YouTube) II. *Hamilton: Love Your City* III. *Intercultural Communication Adventure with Little Pilot* (Dr Deborah Swallow)<br>Knowledge learning: definitions and characteristics of culture<br>b. Self-reflection exercise<br>Activity: Self-reflection questions<br>Video clap: *Who Are You?*<br>Draw your cultural self-portrait<br>Knowledge learning: How culture influences us?<br>Cultural diversity<br>c. Activity: concept matching	90 minutes
Session 2 – Cultural Competence (14:00-17:00 pm)		
Disorienting Dilemma	Video Watching: *The Documentary of HIV/AIDS in China*<br>Small group discussion	40 minutes
Critical Reflection	a. Health and healthcare disparities<br>Knowledge learning: contributing factors and examples of health and healthcare disparities<br>Story: An example of differential care<br>Activity: Identifying your own bias and prejudices<br>Social Attitude Implicit Association Test (SAIAT)<br>b. Cultural competence<br>I. What is cultural competence<br>II. Why cultural competence?<br>c. Cultural scenarios in health care<br>Case discussions: culturally difficult scenarios & culturally sensitive care<br>Why cultural competence matters?<br>Knowledge-centered VS. Skill-centered approaches<br>Story: the risk of solely knowledge-centered approach<br>Tips for transcultural communication	120 minutes
