# Supplementary material for: Increasing undergraduate nursing students’ cultural competence: an evaluation study
Source: Glob Health Res Policy. 2018 Mar 5;3:7. doi: 10.1186/s41256-018-0062-2 (PMC5836416; doi:10.1186/s41256-018-0062-2)
Supplement: Supplementary file 2 — Descriptions of the main teaching materials used in this study. (PDF 510 kb) [file 41256_2018_62_MOESM2_ESM.pdf]

Additional file 2. Descriptions of the main teaching materials used in this study

Intercultural simulation game – *Barnga*

Barnga is a simple card game tournament in which many players can participate. During the game, players are divided into small groups, and they are not informed that each group has been given different rules. After a short orientation, they move from group to group to play the game non-verbally. Cultural shock and conflicts can be simulated because the players tend to assume that every group is playing with the same rules, and it is challenging to comprehend and apply the other rules non-verbally within a short time when participants realize the difference culture (Koskinen, Abdelhamid, & Likitalo, 2008; Graham & Richardson, 2008; Fowler and Pusch, 2010). Compared to *BaFa BaFa*, *Barnga* is less confrontational and nonaggressive, and evokes more feeling of achievement regarding overcoming the cultural conflicts and so on (Koskinen, Abdelhamid, & Likitalo, 2008). An icebreaker at the beginning of the workshop day can put the learners in the right mood to participate in Barnga (Graham & Richardson, 2008). A good facilitator should have the first-hand experience of the game so that he/she can understand the emotional conflicts the participants may suffer. He/she also need to observe the players' reactions and behaviors during the game, and be capable of handling difficult emotions such as conflict, confusion and uncertainty, and try to transform them into a positive experience. Intervention may be needed when the players forget the rules, without disturbing the rhythm of the game. Also, the facilitator needs to promote the learners to connect the simulation and the real world during the debriefing (Graham & Richardson, 2008).

Video

The literature has shown the effects of video clips/ vignettes/DVD in cultural competence education, and various videos have been utilized in intervention, such as *Bridging the Great Divide, Worlds Apart,* and *Unnatural Cause.* Hawala-Druy & Hill (2012) and Lonneman (2015) used documentary *Unnatural Cause: Is Inequality Making Us Sick?* as one of their pedagogical approaches among health professional students and positive effects were found to increase their cultural competence. *Unnatural Causes* is an acclaimed documentary series produced by California Newsreel (Copyright © 2008) and now has been used as an education tool for equity and social justice by thousands of organizations around America. Given the cultural appropriateness, a documentary reflecting the life of vulnerable people living with HIV/AIDS in China (CCTV Official Website, 2011) was used in this study to raise nursing students' awareness of health and health care disparities in China.

Social Attitude Implicit Association Test (SAIAT)

The Social Attitude Implicit Association Test (SAIAT) is a series of online implicit association tests (IAT) about social attitudes designed by Project Implicit. Founded in 1998 by three scientists from University of Washington, Harvard, and Virginia, Project Implicit is a non-profit organization connecting worldwide researchers to create new approaches to explore attitudes, stereotypes, and other hidden biases that effect perception, judgment and behavior. It addresses implicit social cognition, which is defined as "thoughts and feelings outside of conscious awareness and control". Given people do not always be honest to reveal their minds,

some attitudes and beliefs may be reported by this test that people are unwilling to concede or even feel surprised about (Project Implicit, 2011).

The IAT measures the strength of associations between concepts (showed by pictures, e.g., black people, gay people) and evaluations (showed by words, e.g., good, bad) or stereotypes (showed by words, e.g., athletic, clumsy). The main idea is the assumption that matching beliefs with more associated items takes a shorter time. For example, if a person takes longer time to match positive words with pictures of black people than that of white people, then it is assumed that this person prefers white people implicitly and probably holds a more negative attitude towards black people. Thus, one critical point is the person receiving this test must do it as fast as possible, or else the hidden beliefs or attitudes may not be elicited (Steed, 2010; Project Implicit, 2011).

The social attitudes IAT examine the implicit association in the race, age, disability, weight, religion, sexuality, Skin-tone, Gender-science, Gender-Career, Asian American, Native American, Arab-Muslim, Presidents, and Weapons. The implicit preference may not indicate prejudice, but it can predict behavior. When slacking off from the efforts to be egalitarian, the implicit biases can lead to discriminatory behavior, so it is critical to be aware of this possibility to prevent prejudice and discrimination (Project Implicit, 2011).

A meta-analysis of 61 studies (Greenwald, Poehlman, Uhlmann & Banaji, 2009) have found that Implicit Association Test (IAT) measures significantly predicted criterion measures, such as judgments, choices, physiological responses, and behaviors.

The IAT was found to be a procedure that yields reliable and valid measures that predict a side array of meaningful outcomes.

References:

CCTV Official Website (2011). *Eyeshot of Phoenix: The Documentary of HIV/AIDS in China.* Retrieved from http://tv.cntv.cn/video/C32873/7744E7790607419294DEED5DA62CBDB8

Fowler, S. M., & Pusch, M. D. (2010). Intercultural Simulation Games: A Review (of the United States and Beyond). *Simulation & Gaming, 41*(1), 94-115.

Graham, I., & Richardson, E. (2008). Experiential gaming to facilitate cultural awareness: its implication for developing emotional caring in nursing. *Learning in Health & Social Care, 7*(1), 37-46.

Greenwald, A. G., Poehlman, T. A., Uhlmann, E. L., & Banaji, M. R. (2009). Understanding and using the Implicit Association Test: III. Meta-analysis of predictive validity. *Journal of Personality & Social Psychology, 97*(1), 17-41.

Hawala-Druy, S., & Hill, M. H. (2012). Interdisciplinary: Cultural competency and culturally congruent education for millennials in health professions. *Nurse Education Today, 32*(7), 772-779.

Koskinen, L., Abdelhamid, P., & Likitalo, H. (2008). The simulation method for learning cultural awareness in nursing. *Diversity in Health & Social Care, 5*(5), 55-63.

Lonneman, W. (2015). Teaching Strategies to Increase Cultural Awareness in Nursing

Students. *Nurse Educ, 40*(6), 285-288. Doi: 10.1097/NNE.0000000000000175

Project Implicit. (2011). Project Implicit Social Attitudes. [Online Computer Program]. (Available at: https://implicit.harvard.edu/implicit/) (Accessed 24 August 2016).

Steed, R. (2010). Attitudes and beliefs of occupational therapists participating in a cultural competency workshop. *Occup Ther Int, 17*(3), 142-151. doi: 10.1002/oti.299
